# Letter to the Editor: Analysis of stroke patient migration for mechanical thrombectomy and changes in neurointerventional center size in Germany

**DOI:** 10.1186/s42466-021-00131-2

**Published:** 2021-06-07

**Authors:** Ralph Weber, Dirk Bartig, Christos Krogias, Daniel Richter, Werner Hacke, Jens Eyding

**Affiliations:** 1 Department of Neurology, Alfried Krupp Krankenhaus Essen, Alfried-Krupp-Str. 21, 45131 Essen, Germany; 2 Medical Faculty, Ruhr University Bochum, Bochum, Germany; 3 Northwest-German Stroke Circle e.V, Bochum, Germany; 4 drg market, Osnabrück, Germany; 5 Department of Neurology, University Hospital St. Josef-Hospital Bochum, Bochum, Germany; 6 University of Heidelberg, Heidelberg, Germany; 7 Department of Neurology, Gemeinschaftskrankenhaus Herdecke, Herdecke, Germany

**Keywords:** Stroke, Thrombectomy, Health care structure, Patient migration, Neurointerventional center size

## Abstract

**Aim and methods:**

To analyse nationwide changes in neurointerventional center size of all German hospitals performing mechanical thrombectomy (MT) in stroke patients from 2016 to 2019. Furthermore, we assessed cross-district patient migration for MT for the first time using hospitals’ structured quality reports and German Diagnosis-Related Groups data in 2019.

**Findings:**

Number of hospitals performing more than 100 MT procedures/year doubled in Germany from 2016 (*n* = 36) to 2019 (*n* = 71), and these neurointerventional centers performed 71% of all MT procedures in 2019. The overall increase in MT procedures was largely driven by these high-volume neurointerventional centers with ability to perform MT 24/7 (121% increase as compared with 8% increase in hospitals performing less than 100 MT procedures/year). The highest cross-district patient mobility/transfer of stroke patients for MT was observed in districts adjacent to these high-volume neurointerventional centers with existing neurovascular networks.

**Conclusion:**

The substantial increase in MT procedures observed in Germany between 2016 and 2019 was almost exclusively delivered by high-volume stroke centers performing more than 100 MT procedures per year in established neurovascular networks. As there is still a reasonable number of districts with low MT rates, further structural improvement including implementation of new or expansion of existing neurovascular networks and regional tailored MT triage concepts is needed.

## Letter to the Editor

Provision of mechanical thrombectomy (MT) 24/7 for all eligible patients with acute ischemic stroke (AIS) due to large brain vessel occlusion imposes intensive human and technical resource requirements including i.e. possibility to perform multimodal stroke imaging, organization of teleradiology networks, interregional medical service, and organised interplay between regional stroke units and comprehensive stroke centers [1]. We therefore assessed MT volume size of all hospitals and cross-district patient migration for MT in AIS patients in Germany in 2019, as neurovascular networks are continuously emerging and being certified since summer 2017. Nationwide data analysis according to place of residency and place of treatment in AIS patients receiving MT was based upon analysis of the German Diagnosis-Related Groups (G-DRG) data, provided from the Federal Statistical Office (DRG-statistic; www.destatis.de), and the mandatory structured quality reports of all German hospitals (according to §136, 3; Social Code Book V of Germany), which enabled us to assess the numbers of MT procedures performed in each German hospital and patient migration for MT between all 412 German administrative districts and cities. We compared data from 2019 with our last analysis in 2016, in which a detailed description of the methods used can be found [2].

**Fig. 1:**
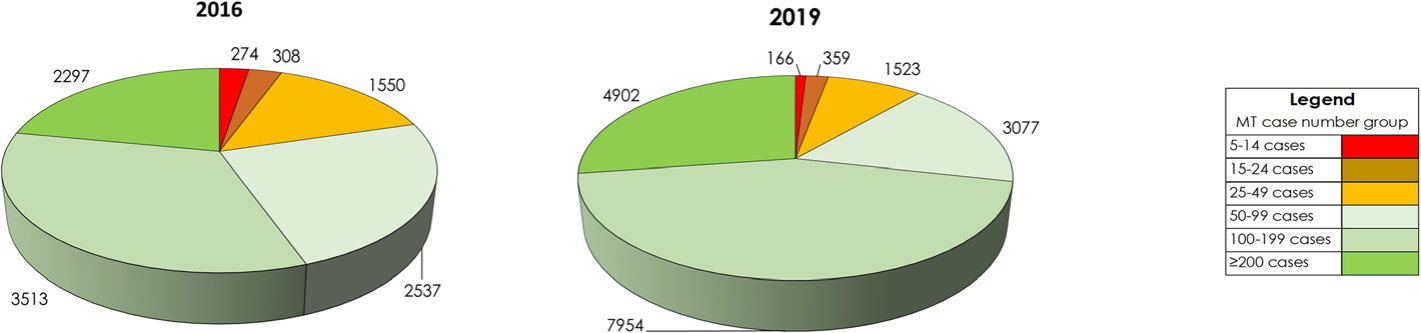
Mechanical thrombectomy volume size of German hospitals 2016 and 2019

In 2019, a total of 190 German hospitals performed five or more MT procedures (OPS code 8–836.80) in patients with admission or in-hospital diagnosis of an AIS based on the structured quality reports, compared to 161 hospitals in 2016. The proportional distribution of MT procedures performed was categorized into seldom (5–14 MT/year), occasional (15–49 MT/year), regular (50–100 MT/year), frequent (100–200 MT/year) and high volume (> 200 MT/year) hospitals (Table 1). Number of hospitals with frequent and high volume neurointerventional centers have doubled in Germany from 2016 (*n* = 36) to 2019 (*n* = 71), and these centers performed almost three quarters (71%) of all MT procedures in 2019 (Table 1 and Fig. 1). The overall increase in MT procedures from 2016 to 2019 was largely driven by hospitals with frequent and high volume neurointerventional centers (121% increase of MT procedures) as compared with an increase of only 8% in hospitals performing less than 100 MT procedures per year (Table 1). The percentage of hospitals performing less than 50 MT procedures per year decreased from 56% in 2016 to 40% in 2019 (Table 1 and Fig. 1).

**Table 1:** Comparison of hospitals performing MT and MT procedure volume between 2016 and 2019

		Number of hospitals (% of all clinics)		Number of MT procedures (% of all MT procedures)	
		2016	2019	2016	2019
**Hospital MT volume**	**5–14 MT/year**	30 (18.5%)	16 (8%)	274 (2.5%)	166 (1%)
	**15–24 MT/year**	16 (10%)	19 (10%)	308 (3%)	359 (2%)
	**25–49 MT/year**	44 (27%)	41 (22%)	1550 (15%)	1523 (9%)
	**50–99 MT/year**	35 (22%)	43 (23%)	2537 (24%)	3077 (17%)
	**100–199 MT/year**	27 (17%)	56 (29%)	3513 (33.5%)	7954 (44%)
	**> 200 MT/year**	9 (5.5%)	15 (8%)	2297 (22%)	4902 (27%)

**Fig. 2:**
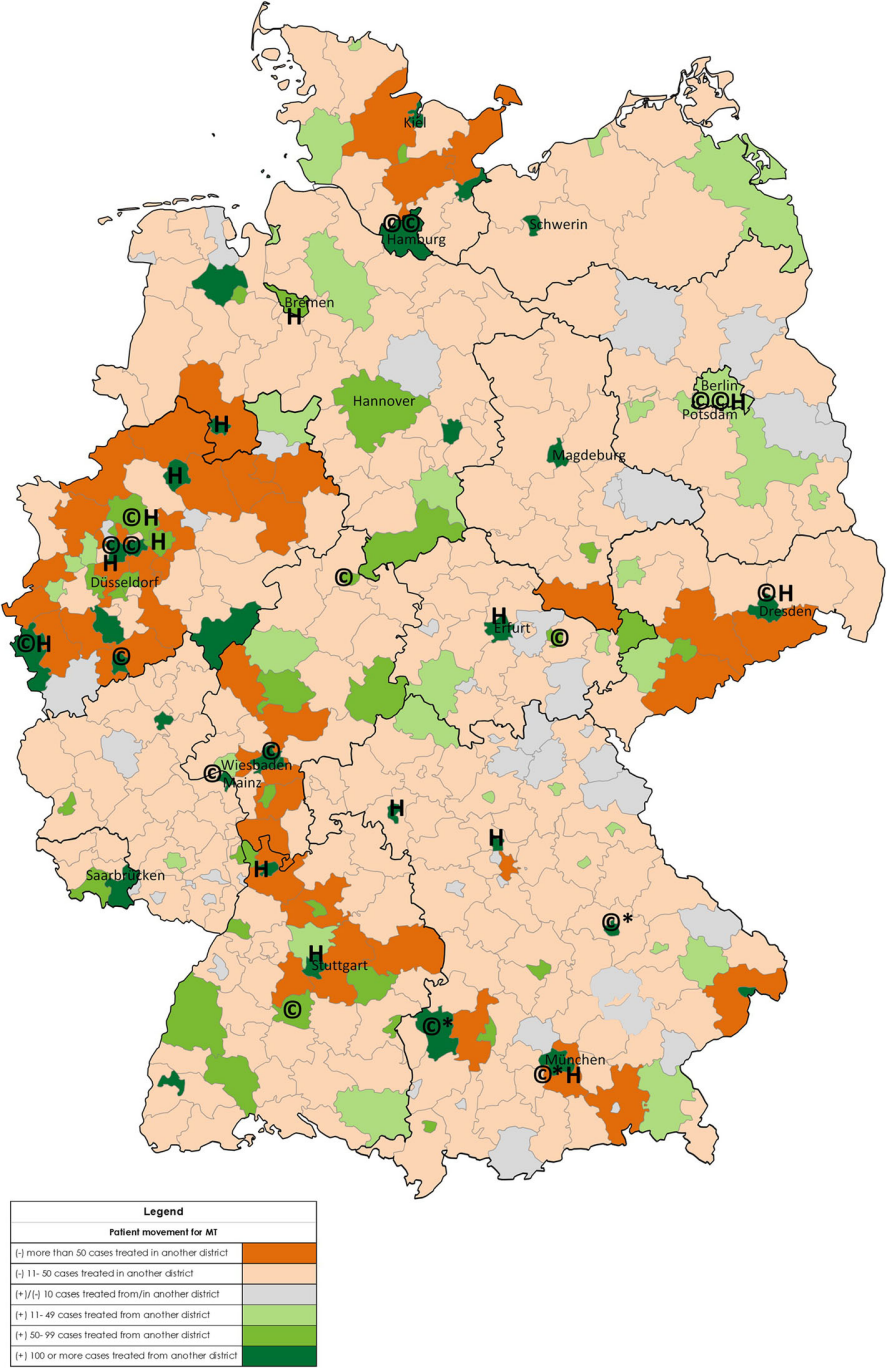
Cross-district patient migration for MT in Germany in 2019. Certified neurovascular networks as indicated by the German Stroke Society in April 2021 are indicated with “C” and high-volume neurointerventional centers (> 200 MT/year) are indicated with “H”. The neurovascular network in southern Bavaria with its 3 geographical locations has been marked with *. State capitals of all 16 German federal states are named

In 2019, the greatest patient migration for MT was observed in districts adjacent (dark orange in Fig. 2) to frequent/high volume neurointerventional centers (dark green cities/districts in Fig. 2) at university hospitals and city hospitals with distinct neurovascular networks in the following regions: Northern Germany (Kiel/Lübeck/Hamburg), Region Münster/Osnabrück, Ruhr region, Rhineland, Region Siegen/Marburg/Gießen, Rhein-Main region, Neckar region, 4 regions in Bavaria (Augsburg/Günzburg, Munich, Passau, Erlangen), and Saxonia (Dresden/Chemnitz/Zwickau/Altenburg). Of note, German’s largest city Berlin with its ‘closed system’ of multiple stroke units, neurovascular centers and networks has to be considered separately.

Our data clearly show that the substantial increase in MT procedures in Germany from 2016 to 2019, driven by implementation of MT in stroke guidelines and expanded time windows and indications for MT [3, 4], was almost exclusively delivered by comprehensive stroke hospitals performing more than 100 MT procedures/year who have the possibility of providing MT 24/7. Higher rates of favorable outcome have been observed in such high volume comprehensive stroke centers [5], and an increasing interhospital transfer for MT has also been reported from other countries [6]. Thus, the observed trend of a decreasing number of hospitals performing MT procedures only seldom or occasionally from 2016 to 2019 is a welcome development.

On the other hand, AIS patients in 260 German districts/district free cities still had no direct access to a hospital performing MT in 2019 (271 in 2016), and cross-district patient migration for MT was substantially higher in districts directly adjacent to hospitals performing MT on a frequent or high volume level, resulting in a continuous unequal access to and/or substantial longer transfer distances in AIS patients eligible for MT. This finding is most likely the main reason for the observed high regional variability of MT rates (from 1.4 to 15.2%) in German AIS patients in 2019 [7], and warrant further structural improvement including implementation of more neurovascular networks and regional tailored triage concepts of providing MT [8].

## Data Availability

Availability of data is outlined in the methods section: 1) DRG-statistic, Federal Statistical Office, www.destatis.de; 2) structured Quality Reports of hospitals according to §136 3.1 No. 4 SGB V (reported years 2016 and 2019), XML-version, G-BA, Quality Reports of the hospitals are used partially and are combined with other sources, the specified recommendations and insights are not to be named as authentic reproduction of the Quality Reports, the complete and unaltered insights of the Quality Reports are to be found via www.g-ba.de.
